# Understanding Age-Based Differences in Psychosocial Harms, Physical Harms, and Access Difficulties Among an International Sample of Men Who Use Anabolic-Androgenic Steroids

**DOI:** 10.1177/15598276251405211

**Published:** 2025-12-18

**Authors:** Benjamin Bonenti, Vigorous Steve, Cheneal Puljević, Jason Ferris, Monica J. Barratt, Adam Winstock, Lauren Ball, Merryn Armstrong, Timothy Piatkowski

**Affiliations:** 1 School of Applied Psychology, Griffith University, QLD, Australia; 2 Centre for Health Services Research, 1974 The University of Queensland, Brisbane, QLD, Australia; 3 Independent Researcher, Bangkok, Thailand; 4 School of Public Health, 1974 The University of Queensland, Brisbane, QLD, Australia; 5 Social Equity Research Centre and Digital Ethnography Research Centre, 5376 RMIT University, Melbourne, VIC, Australia; 6 National Drug and Alcohol Research Centre, UNSW Sydney, Sydney, NSW, Australia; 7 Institute of Epidemiology and Health Care, University College, London, UK; 8 Global Drug Survey, London, UK; 9 Centre for Community Health and Wellbeing, 1974 The University of Queensland, Brisbane, QLD, Australia; 10 School of Human Movement and Nutrition Sciences, 1974 The University of Queensland, Brisbane, QLD, Australia; 11 Mayhem Strong, Brisbane, QLD, Australia; 12 Queensland Injectors Voice for Advocacy and Action, Sunshine Coast, QLD, Australia

**Keywords:** anabolic-androgenic steroids, psychosocial harms, physical harms, access difficulties, age differences

## Abstract

**Background:** Anabolic-androgenic steroid (AAS) use is rising globally. However, age-related differences in harms and health care access barriers remain underexplored. As such, this study examined age-based differences in AAS-related harms and access to support services. **Methods:** The sample (N = 1146) comprised men who reported AAS use within the prior 12 months, stratified into 2 age groups (<40 years and ≥40 years). Chi-square analyses were conducted to examine age-related differences in physical health concerns, psychosocial issues, and barriers to health care access. UpSet plots were used to visualise co-occurrence patterns within each domain. **Results:** Younger men who use AAS were significantly more likely than older men who use AAS to report psychosocial concerns, including anger and depression (all *χ*^*2*^s > 4.0, all *p*s < .045). They also reported higher rates of physical concerns, including hair loss and fertility issues (all *χ*^*2*^s > 6.3, all *p*s < .012). Access difficulties were also more prevalent among the younger men (*χ*^*2*^s > 4.0, *p*s < .046), particularly with pharmacies and hospitals. **Conclusions:** Younger men who use AAS appear to face greater burden and complexity than older men who use AAS, underscoring the need for targeted harm reduction strategies and improved service engagement pathways tailored to peoples’ age.

“Older men who use AAS may be more likely to receive prescriptions or assistance from clinicians, particularly when presenting with age-related testosterone decline”.

## Introduction

Anabolic-androgenic steroids (AAS) are increasingly consumed off-label,^1,2^ with an estimated 3.3% of the global population having used AAS at least once. Men account for the majority of people who use AAS at 6.4%,^3^ compared to 4% among women.^4^ While AAS were originally developed to address conditions such as hypogonadism^5^ and muscle-wasting diseases,^6^ contemporary AAS use is overwhelmingly driven by non-medical motivations.^7,8^ For instance, younger men seek aesthetic and performance-related outcomes, such as increased muscularity, reduced body fat, and improved recovery.^7-9^ Such motives shape risk-taking behaviours (e.g. decisions around dosage and administration), access pathways (e.g. procurement through unregulated peer markets), and patterns of AAS type selection.^10,11^ In contrast, older men have historically accessed AAS through medically supervised hormone replacement therapy (HRT), primarily to restore declining hormone levels, and alleviate symptoms of hypogonadism.^12-14^ However, emerging evidence suggests that even older men are increasingly drawn into using AAS for enhancing their performance and wellbeing, going well beyond therapeutic HRT doses.^15-17^ Qualitative evidence similarly points to age-coded attitudinal differences: younger men emphasise power/control and appearance-driven ideals, whereas older men more often frame AAS use in athletic/performance terms.^18^ Despite the well-documented^19-25^ harms (e.g. cardiomyopathy)^19-25^ associated with AAS use, the extent to which these harms differ by age remains unexamined. This study addresses this critical unknown, presenting a comparative profile of psychosocial concerns, physical concerns, and health care service access difficulties reported by younger and older men who use AAS.

AAS consumption spans a wide demographic, whereby most people who use AAS – regardless of age – are motivated by a desire to enhance muscularity, reduce fat mass, and improve physical performance.^7,8^ These goals reflect a broad cultural shift away from therapeutic, medically sanctioned use, toward non-medical, physique-driven consumption.^15^ For younger men who use AAS, there is a heavy reliance on informal online and offline peer networks that dispense ethnopharmacological (also known as ‘bro-science’)^26^ advice in the absence of mainstream medical support.^11,27,28^ This disparity often results in sourcing compounds through unregulated channels, contributing to a range of access-related risks.^11,27^ This trend is fuelled by widespread stigma among health care providers,^29,30^ and a lack of health care practitioner literacy regarding AAS-related harm reduction,^31^ leaving many younger men excluded from evidence-based care.^9,11^ In contrast, older men have historically engaged with AAS via clinically supervised HRT, granting them greater access to medical oversight, reliable compound sources, and safer administration techniques.^12,14,32^ Their health-oriented use motives may also reduce the likelihood of experiencing access barriers, enabling more stable and regulated usage.^14,32,33^ However, emerging evidence suggests that this distinction is narrowing, with older people who use AAS increasingly adopting appearance and performance-driven motives that mirror their younger counterparts.^17^ Despite these shifting trajectories, no research to date has systematically examined how age may shape the burden and complexity of AAS-related harms and access difficulties.

Understanding how AAS-related harms manifest across the lifespan is critical, given the well-established risks associated with AAS consumption. From a physiological standpoint, excessive or prolonged androgen exposure, particularly under self-directed protocols,^34^ has been strongly linked to cardiovascular pathology, including left ventricular hypertrophy, atherosclerotic changes, hypertension, and dyslipidaemia.^19-22^ These risks are amplified by using unregulated compounds,^35^ incorrect administration techniques, and unsafe injecting practices, which can introduce additional complications, such as bacterial infections, abscess formation and blood-borne viruses (e.g. hepatitis C, HIV).^36,37^ However, it remains unclear whether these harms are distributed evenly across age groups, or whether younger people who use AAS – by virtue of their risk-taking behaviours – face a heavier burden.^10^ In addition to physical risks, psychosocial complications are also consistently reported among people who use AAS, with studies documenting elevated rates of irritability, aggression, anxiety, depressive symptoms, cognitive dysfunction, and impulse dysregulation,^23-25^ particularly in the context of high-dose or prolonged consumption.^38,39^ These outcomes often emerge from the same cluster of vulnerabilities underpinning physical risk, non-medical use,^7^ low health literacy,^40^ restricted access to regulated compounds,^41^ and limited access to clinical oversight.^11,27^ Clarifying how these risk profiles differ between younger and older people who use AAS, is therefore, essential for developing age-targeted harm reduction strategies, and promoting safer AAS practices across the lifespan for optimal health and social outcomes.

Age stratification is particularly important given evidence that serum testosterone levels remain relatively stable across the 20s and 30s but begin to decline from approximately age 40 – at a rate of around 0.4% per year for total testosterone and approximately 1.3% annually for free testosterone.^42^ This decline marks a biological and clinical shift in male endocrine function, representing a transition from youthful hormonal baselines to age-related hypogonadism in many men.^5,43^ As such, younger men (aged <40) are more likely to engage in non-medical AAS use to enhance appearance and performance, typically through unmonitored cycles and peer-informed protocols.^17,42,44^ In contrast, older men (aged ≥40) are more likely to use AAS to self-treat declining testosterone associated with age-related hypogonadism – commonly referred to as late-onset hypogonadism – though this distinction is increasingly blurred.^17,43^ This study’s focus on cisgender men aligns with longstanding clinical patterns in testosterone therapy, where hypogonadism in men has been the primary medical indication until recent years, when gender-affirming care began gaining formal recognition.^5,45^

Therefore, the present study aimed to clarify age-based differences in the physical concerns, psychosocial concerns, and health care service access difficulties of men who use AAS. Using data from the 2024 Global Drug Survey (GDS2024), this study compares younger (<40) and older (≤40) men who use AAS, to identify age-based differences in the burden and configuration of AAS-related harms and access difficulties.

## Method

### Sample and Procedure

Data for this study were drawn from the GDS2024, an annual, anonymous online survey designed to examine patterns of substance use worldwide. Participants were recruited through a combination of legacy media outlets (e.g. *The Sydney Morning Herald*), major social media platforms (e.g. X [formerly Twitter], YouTube), and direct engagement by social media influencers (e.g. Vigorous Steve). The survey captures a range of sociodemographic variables, along with detailed information on participants’ drug consumption histories and related outcomes. GDS2024 was active from 10 January to 30 April 2024, and required between 15 and 60 min to complete, depending on the individual’s drug consumption profile. Comprehensive information on the survey’s development, recruitment strategy, and validation procedures is available elsewhere.^46-48^

Eligibility criteria for participation included being at least 16 years of age and reporting the consumption of at least 1 psychoactive substance in the past 12 months. The survey was fully anonymous, with no collection of IP addresses or any personally identifying information such as names, dates of birth, or residential addresses. Participants were informed that the purpose of the research was to better understand global patterns of alcohol and drug use to inform harm reduction and public health strategies. While recruitment strategies ensured wide international reach, the sample remains a large convenience sample rather than a probability sample, and representativeness across all regions cannot be assumed.

### Measures

The present study utilised demographic information pertaining to age, gender, country of residence, highest education level and occupation. Further, questions were asked about participants’ AAS usage, including whether they have consumed AAS within the previous 12 months, their age when first trying injectable AAS, oral AAS consumption (lifetime and prior 12 months), and injectable AAS consumption (lifetime and prior 12 months). The GDS instrument is developed annually by an international team of researchers, clinicians, and harm reduction experts, with the intent of mapping global patterns of alcohol and other drug use. Items are iteratively refined through pilot testing and expert consultation, and the instrument has been extensively employed across more than 30 countries. Its validity and reliability for assessing substance use behaviours and related outcomes have been demonstrated in multiple peer-reviewed publications.^46-48^

The study also gathered data relating to the psychological and physical concerns associated with each AAS usage strategy. Example items include ‘physical concern: Hair loss’, and participants were required to select ‘yes’ or ‘no’ for each. Specifically, the *psychological concerns* section included items pertaining to the following concerns: anger/aggression, depression/low mood, rapid fluctuation in mood, and restlessness/irritability. The *physical concerns* section included items assessing concerns pertaining to decreased sexual function, hair loss, negative impacts on sexual organs, decreased fertility, skin condition (e.g. acne), and negative impact on heart. The *access* section included items assessing the following health care services: needle service providers, general practitioners, hospitals, and pharmacies. For psychological, physical, and access concerns an open-ended ‘other’ option was also available to allow participants to provide more details. These items were presented in a dichotomous format (yes/no), consistent with GDS design principles, to maximise clarity and reduce response burden in a large international survey. Prior waves of the GDS^46^ have routinely employed binary health, harm, and behaviour indicators, providing direct methodological precedent for the present wave. This approach is also reflected in AAS analyses conducted within the GDS framework, including recent work examining clenbuterol-related harms^49^ and broader AAS health-monitoring patterns,^50^ where dichotomous variables support reliable reporting in community-based samples. Collectively, this framework balances interpretive clarity with practical feasibility in high-volume international datasets.

#### Analysis

All analyses were conducted using SPSS Version 30^51^ and, all graphics were created using Python Version 3.12^52^. The primary analysis examined differences in psychosocial concerns, physical concerns, and health care access between individuals aged <40 years old (the <40 group), and individuals aged ≥40 years old (the ≥40 group). This age division (<40 vs ≥40) was selected based on well-documented endocrine shifts beginning around age 40.^5,42,43^ Chi-square tests of independence were conducted to determine whether the prevalence of individual psychosocial concerns, physical concerns, and access difficulties differed between age categories. Phi (*ϕ*) coefficients were reported as effect sizes, with *ϕ* = .10, .30, and .50 interpreted as small, medium, and large effects, respectively.^53^ To examine the distribution and overlap of concerns, multiple response frequency tables were generated for psychosocial concerns, physical concerns, and access difficulties displaying the proportion of responses and cases reporting *yes* for each. Participants could select multiple concerns perceived as resulting from their AAS consumption, across psychosocial (8 items; 255 possible response combinations), physical (12 items; 4095 combinations), and access difficulty domains (5 items; 31 combinations), respectively. Further, the total number of concerns reported per participant was computed, yielding the psychosocial concern pattern (the number of psychosocial concerns each participant endorsed [0 to 8]), physical concern pattern (the number of physical concerns each participant endorsed [0 to 11]), and access difficulties pattern (the number of access difficulties each participant endorsed [0 to 6]).

The concern/access variables were binary, whereby a score of 0 or 1 indicated the participant did not or did indicate (respectively) that particular concern. The tables illustrate the extent to which participants experience zero, isolated, or multiple psychosocial side effects, physical side effects, and access difficulties. Additionally, UpSet plots were generated to visualise the co-occurrence of psychosocial concerns, physical concerns, and access difficulties across age groups. These plots illustrate the intersections and sizes of multiple response sets, providing insight into the co-occurrence (and frequency) of symptoms in each age group. Separate UpSet plots were produced separately for the <40 and ≥40 groups across psychosocial concerns, physical concerns, and access difficulties. For all analytical tests, a *p*-value of <0.05 was considered statistically significant. Assumptions for chi-square tests were met, as all expected cell frequencies were >5 in at least 80% of cases.

## Results

### Descriptive Statistics

#### Demographic Characteristics

Data were drawn from 12 615 participants from 117 countries (2615 participants did not provide country data) who responded to the GDS2024, completed the image and performance enhancing drug (IPED) module and self-identified as men (N = 1146, *M*_*age*_ = 31.46, *SD* = 9.93) with a self-reported history of AAS consumption within the past 12 months and were aged either <40 (n = 920, *M*_*age*_ = 27.58) or ≥40 (n = 226, *M*_*age*_ = 47.25). Table 1 and Table 2 show the frequencies (for categorical demographic characteristics) and descriptive statistics (for continuous demographic characteristics) of the overall sample, respectively.

**Table 1.** Frequencies for the Categorical AAS Usage Characteristics of the Overall Sample (N = 1146), and Participants Who are Aged <40 (n = 920) and ≥40 (n = 226).

Characteristic	Category		Frequency (%)	
		Overall sample	<40	≥40
Lifetime consumption of injectable steroids	Yes	840 (73.3%)	660 (71.7%)	180 (79.6%)
	No	306 (26.7%)	260 (28.3%)	46 (20.4%)
Consumption of injectable steroids in last 12 months	Yes	774 (67.5%)	609 (66.2%)	165 (73.0%)
	No	372 (32.5%)	311 (33.8%)	61 (27.0%)
Lifetime consumption of oral steroids	Yes	728 (63.5%)	578 (62.8%)	150 (66.4%)
	No	418 (36.5%)	342 (37.2%)	76 (33.6%)
Consumption of oral steroids in last 12 months	Yes	538 (46.9%)	441 (47.9%)	97 (42.9%)
	No	608 (53.1%)	479 (52.1%)	129 (57.1%)

**Table 2.** Descriptive Statistics for the Continuous AAS Usage Characteristics of the Overall Sample (N = 1146), and Participants Who are Aged <40 (n = 920) and ≥40 (n = 226).

Characteristic	M(SD)		
	Overall sample	Under 40	Over 40
Age	31.46 (9.93)	27.58 (6.00)	47.25 (6.54)
Age first consumed injectable AAS	26.24 (7.66)	24.05 (5.23)	33.19 (10.30)
Age first consumed oral AAS	26.60 (8.02)	24.34 (5.29)	34.48 (10.79)

For the categorical demographic variables, there was a significant difference between the age groups for lifetime consumption of injectable steroids, *χ*[1] = 5.80, *p* = .016, whereby the ≥40 group were significantly more likely to have consumed injectable steroids in their lifetime (79.6%) compared to the <40 group (71.7%). There was also a marginally significant difference between the age groups for consumption of injectable steroids within the last 12 months, *χ*[1] = 3.84, *p* = .050, whereby the ≥40 group were slightly more likely to have consumed injectable AAS within the last 12 months (73.0%) than the <40 group (66.2%). In contrast, there was a non-significant difference across the age groups for lifetime consumption of oral AAS, *χ*[1] = 0.98, *p* = .321. Participants <40 reported a slightly lower prevalence of oral steroid consumption in their lifetime (62.8%) compared to those ≥40 (66.4%), but this difference was not statistically significant. Finally, there was a non-significant difference across the age groups for consumption of oral steroids within the last 12 months, *χ*[1] = 1.83, *p* = .176, whereby the <40 group and ≥40 group reported similar rates of oral AAS consumption within the last 12 months (47.9% and 42.9%, respectively).

For the continuous demographic variables, there was a significant difference between the age groups in chronological age, *t* (1144) = −43.34, *p* < .001, with the ≥40 group being significantly older (*M* = 47.25) than the <40 group (*M* = 27.58). There were also highly significant differences between age groups, in terms of age at first using injectable steroids, *t* (818) = −16.17, *p* < .001, and for age at first using oral steroids, *t* (699) = −16.04, *p* < .001. Specifically, the ≥40 group reported initiating consumption of injectable AAS at an older age (*M* = 33.19) compared to the <40 group (*M* = 24.05) and similarly reported a later age of first oral AAS consumption (*M* = 34.48) compared to the <40 group (*M* = 24.34).

#### Psychosocial Concerns

Participants selected a total of 1071 concerns, indicating frequent endorsement of multiple concerns per individual (see Table 3). The most frequently reported concerns were restlessness/irritability (17.4% of all responses), followed by depression/low mood (15.4%) and rapid fluctuation in mood (13.3%). The least frequently reported concerns were relationship difficulties (11.0%) and other unspecified concerns (6.2%).

**Table 3.** Multiple Responses for Psychosocial Concerns for Participants (N = 1146) Who are Aged <40 (n = 920) and *≥*40 (n = 226).

Psychosocial Concern	Frequency	% of Responses	% of Cases
Restlessness/irritability	186	17.4	16.2
Depression/low mood	165	15.4	14.4
Rapid fluctuation in mood	142	13.3	12.4
Irrational excitability/elevation of mood	132	12.3	11.5
Anger/aggression	134	12.5	11.7
Loss of interest in other things	128	11.9	11.2
Quality of my relationship with others	118	11	10.3
Other	66	6.2	5.8
Total	1071	100	

A total of 55.8% (n = 639) of participants reported no psychosocial concerns, while the remaining 44.2% endorsed at least 1 concern. Among those reporting concerns, 21.5% (n = 246) reported only 1, while 9.9% (n = 114) reported 2 concerns. As the number of concerns increased, fewer participants endorsed them, with 5.9% (n = 68) reporting 3 and 3.2% (n = 37) reporting 4. A small subset endorsed 5 (1.7%, n = 19), 6 (1.0%, n = 12), or 7 (0.9%, n = 10) concerns, while only 1 person endorsed all 8 concerns (see Table 4 for full breakdown).

**Table 4.** Psychosocial Concerns Pattern Table Displaying the Number of Participants (N = 1146) Reporting Each Psychosocial Concern.

Number of psychosocial concerns	Frequency	Percent	Cumulative percent
0	639	55.8	55.8
1	246	21.5	77.3
2	114	9.9	87.2
3	68	5.9	93.1
4	37	3.2	96.3
5	19	1.7	98
6	12	1	99
7	10	0.9	99.9
8	1	0.1	100
Total	1146	100	

##### Overlap: UpSet Plots

**Figure 1.**
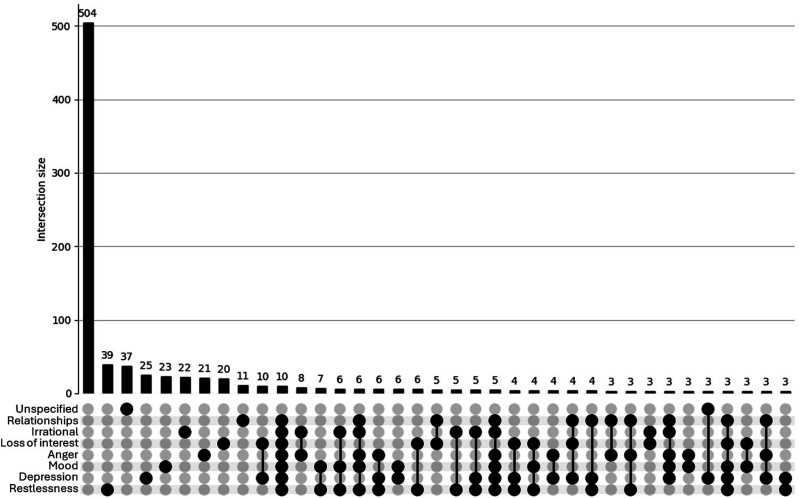
UpSet plot depicting psychosocial concerns for participants (n = 920) aged <40. Footnote: Unspecified = other/unspecified; Relationships = Quality of my relationship with others; Irrational = Irrational excitability/elevation of mood; Loss of interest = Loss of interest in other things; Anger = Anger/aggression; Mood = Rapid fluctuation of mood; Depression = Depression/low mood; Restlessness = Restlessness/irritability. For brevity, combinations of <3 concerns are not shown in this plot.

**Figure 2.**
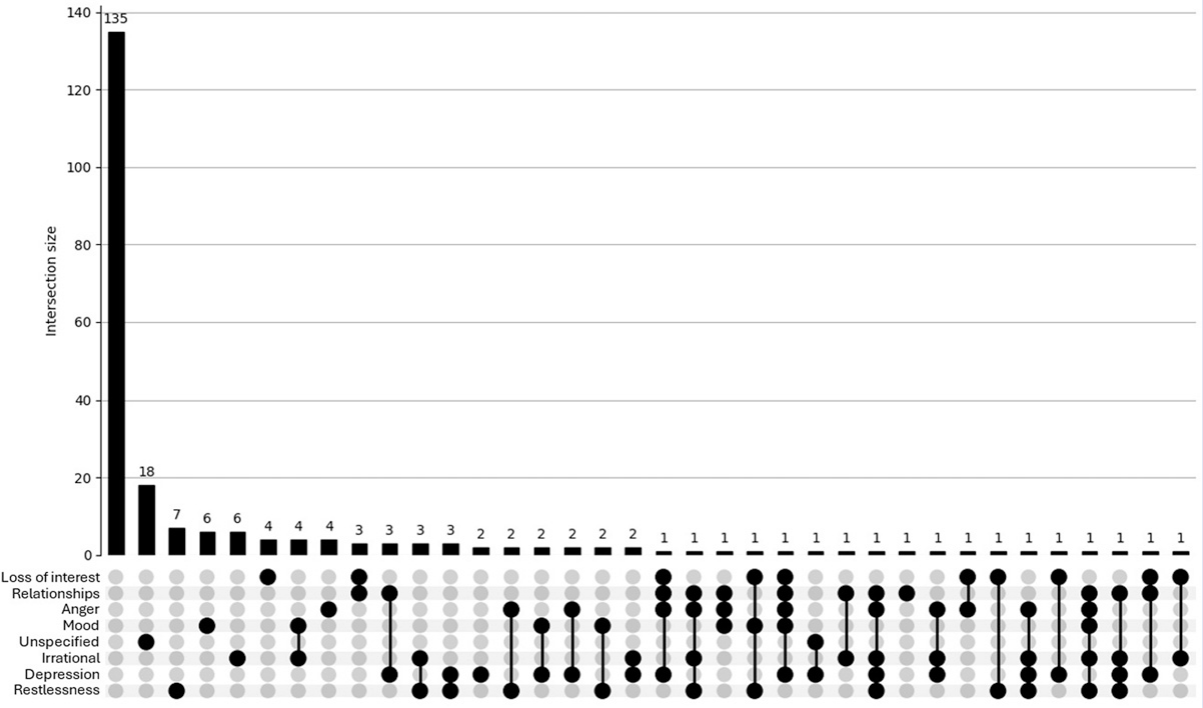
UpSet plot depicting psychosocial concerns for participants (n = 226) aged *≥*40. Footnote: Unspecified = other/unspecified; Relationships = Quality of my relationship with others; Irrational = Irrational excitability/elevation of mood; Loss of interest = Loss of interest in other things; Anger = Anger/aggression; Mood = Rapid fluctuation of mood; Depression = Depression/low mood; Restlessness = Restlessness/irritability.

The UpSet plot for the <40 group (see Figure 1) reveals a densely populated distribution of psychosocial concerns. While a large proportion of participants reported no concerns (n = 504), there is a notable presence of overlapping concerns, with numerous intersections involving 2 or more symptoms (e.g. anger, restlessness, depression). This pattern suggests a higher burden of co-occurring psychosocial issues among younger participants, reflecting a psychosocial profile that would likely benefit from health care or other support. In contrast, the UpSet plot for the ≥40 group (see Figure 2) displays a simpler distribution. Most individuals reported either no concerns (n = 135) or only single symptoms, with relatively few instances of overlapping issues. Although some intersections involving 2 or 3 concerns are present, these are less frequent and less pronounced. This pattern suggests a comparatively lower burden and complexity of psychosocial side effects in the older age group.

#### Physical Concerns

Participants selected a total of 2415 concerns, indicating frequent endorsement of multiple concerns per individual (see Table 5). The most frequently reported concerns were negative impact on the heart (17.1%), hair loss (14.6%), and negative impact on the liver (12.6%). The least frequently reported concerns were reduction in breast size (0.2%) and increase in breast size (1.6%).

**Table 5.** Multiple Responses for Physical Concerns for Participants (N = 1146) Who are Aged <40 (n = 920) and *≥*40 (n = 226).

Physical concern	Frequency	% of Responses	% of Cases
Decreased sexual function	196	8.1	17.1
Hair loss	352	14.6	30.7
Hair gain	113	4.7	9.9
Negative impact(s) on sexual organs	150	6.2	13.1
Decreased fertility	235	9.7	20.5
Growth of breasts (in males only)	264	10.9	23
Reduction in breast size	5	0.2	0.4
Increase in breast size	39	1.6	3.4
Skin condition (e.g. acne)	299	12.4	26.1
Negative impact on heart	413	17.1	36
Negative impact on liver	304	12.6	26.5
Other	45	1.9	3.9
Total	2415	100	

A total of 36.1% (n = 414) of participants reported no physical concerns, while the remaining 63.9% endorsed at least 1 concern. Among those reporting concerns, 13.6% (n = 156) reported only 1, while 14.1% (n = 162) reported 2 concerns. As the number of concerns increased, fewer participants endorsed them, with 12.0% (n = 138) reporting 3 and 7.5% (n = 86) reporting 4. A small subset endorsed 5 (6.2%, n = 71), 6 (5.1%, n = 58), 7 (2.6%, n = 30), 8 (1.7%, n = 20), or 9 concerns (0.8%, n = 9). Very few participants reported 11 or 12 concerns (0.1% each, n = 1). See Table 6 for full breakdown.

**Table 6.** Physical Concerns Pattern Table Displaying the Number of Participants (N = 1146) Reporting Each Psychosocial Concern.

Number of physical concerns	Frequency	Percent	Cumulative percent
0	414	36.1	36.1
1	156	13.6	49.7
2	162	14.1	63.8
3	138	12	75.8
4	86	7.5	83.3
5	71	6.2	89.5
6	58	5.1	94.6
7	30	2.6	97.2
8	20	1.7	98.9
9	9	0.8	99.7
11	1	0.1	99.8
12	1	0.1	99.9
Total	1146	100	

##### Overlap: UpSet Plots

**Figure 3.**
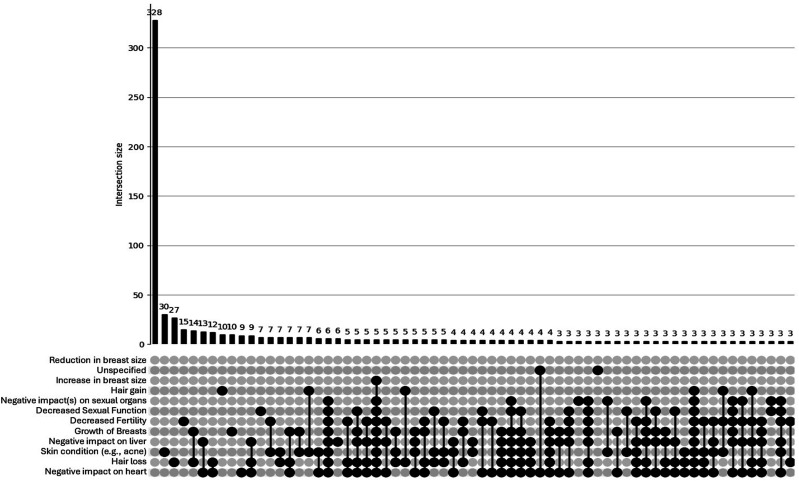
UpSet plot depicting physical concerns for participants (n = 920) aged <40. Footnote: Breast reduction = Reduction in breast size; Breast increase = Increase in breast size; Sexual organs = Negative impact(s) on sexual organs; Sexual function = Decreased sexual function; Skin = Sin condition (e.g. acne); Breast growth = Growth of breasts (in males only); Liver = Negative impact on liver; Heart = Negative impact on heart. For brevity, combinations <3 concerns are not shown in this plot.

**Figure 4.**
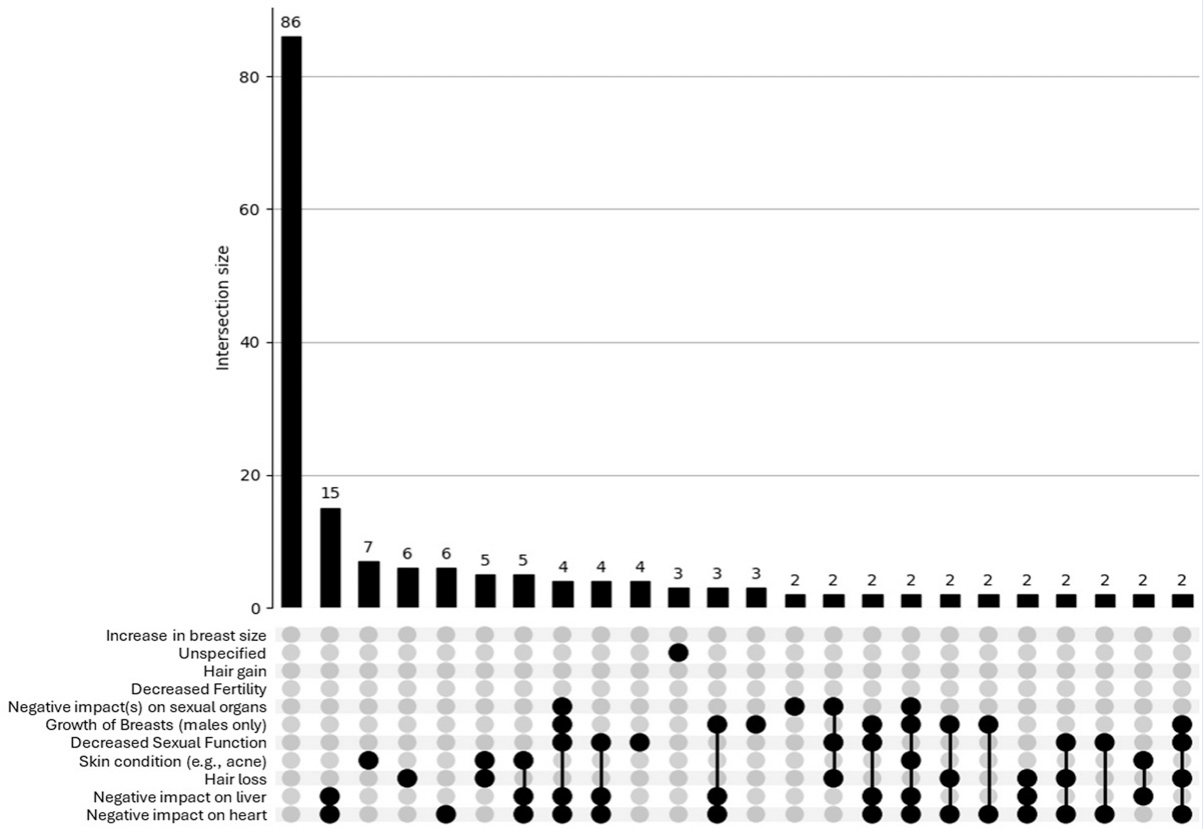
UpSet plot depicting physical concerns for participants (n = 226) aged *≥*40. Footnote: Breast reduction = Reduction in breast size; Breast increase = Increase in breast size; Sexual organs = Negative impact(s) on sexual organs; Sexual function = Decreased sexual function; Skin = Sin condition (e.g. acne); Breast growth = Growth of breasts (in males only); Liver = Negative impact on liver; Heart = Negative impact on heart. For brevity, combinations <2 concerns are not shown in this plot.

The UpSet plot for the <40 group (see Figure 3) shows a broad and intricate distribution of physical concerns. While a large proportion reported no concerns (n = 328), there is a substantial number of participants reporting overlapping side effects, including numerous intersections involving 2 or more physical concerns (e.g. hair loss, fertility issues, cardiovascular strain). This pattern indicates a more complex and multifaceted side-effect profile among younger men who use AAS, also – like psychosocial concerns – reflecting a psychosocial profile that would likely benefit from health care or other support. In contrast, the UpSet plot for the *≥*40 group (see Figure 4) reveals a more limited pattern of physical side effects. Although some overlap is present, most intersections involve only 1 or 2 concerns, and higher-order overlaps are sparse. The overall distribution is flatter and less dense, suggesting that older participants tend to report fewer and less interconnected physical side effects.

#### Access Difficulties

Participants selected a total of 466 barriers to accessing health care (see Table 7). The most frequently reported difficulties were with general practitioners (49.1% of all responses), followed by pharmacies (18.7%) and hospitals (14.8%). The least frequently reported difficulties were with needle service providers (10.1%) and other unspecified services (7.3%).

**Table 7.** Multiple Responses for Access Difficulties for Participants (N = 1146) Who are Aged <40 (n = 920) and *≥*40 (n = 226).

Access difficulty	Frequency	% of Responses	% of Cases
Needle service providers	47	10.1	4.1
General practitioners	229	49.1	20
Hospitals	69	14.8	6
Pharmacies	87	18.7	7.6
Other	34	7.3	3
Total	466	100	

A total of 46.2% (n = 530) of participants reported no difficulties in accessing health services for their AAS consumption, while the remaining 53.8% endorsed at least 1 difficulty. Among those reporting difficulties, 0.3% (n = 3) reported only 1, while 13.2% (n = 151) reported 2. As the number of difficulties increased, fewer participants endorsed them, with 6.5% (n = 74) reporting 3 and 2.6% (n = 30) reporting 4. A small subset endorsed 5 (1.6%, n = 18) or 6 difficulties (0.1%, n = 1). See Table 8 for full breakdown.

**Table 8.** Pattern Table Displaying the Number of Participants (N = 1146) Reporting Each Access Difficulty.

Number of access difficulties	Frequency	Percent	Cumulative percent
0	530	65.7	65.7
1	3	0.4	66.1
2	151	18.7	84.8
3	74	9.2	94
4	30	3.7	97.7
5	18	2.2	99.9
6	1	0.1	100
Total	807	100	

##### Overlap: UpSet Plots

**Figure 5.**
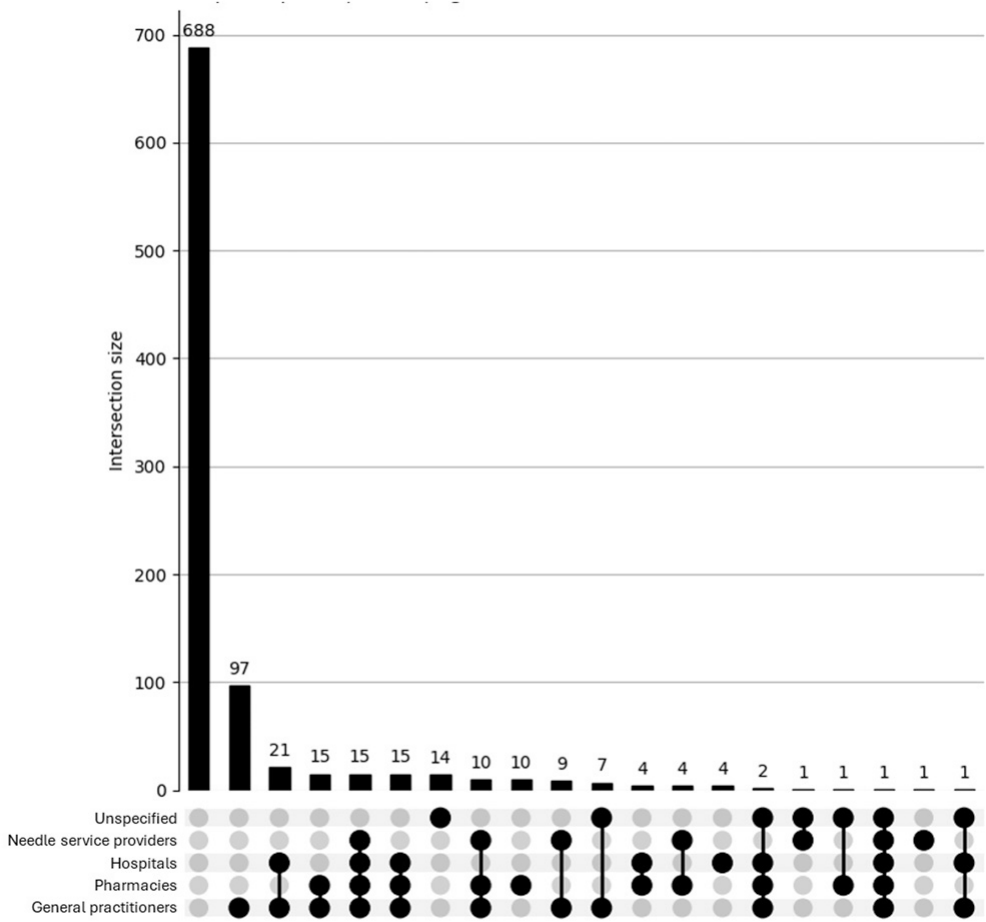
UpSet plot depicting access difficulties for participants (n = 920) aged <40.

**Figure 6.**
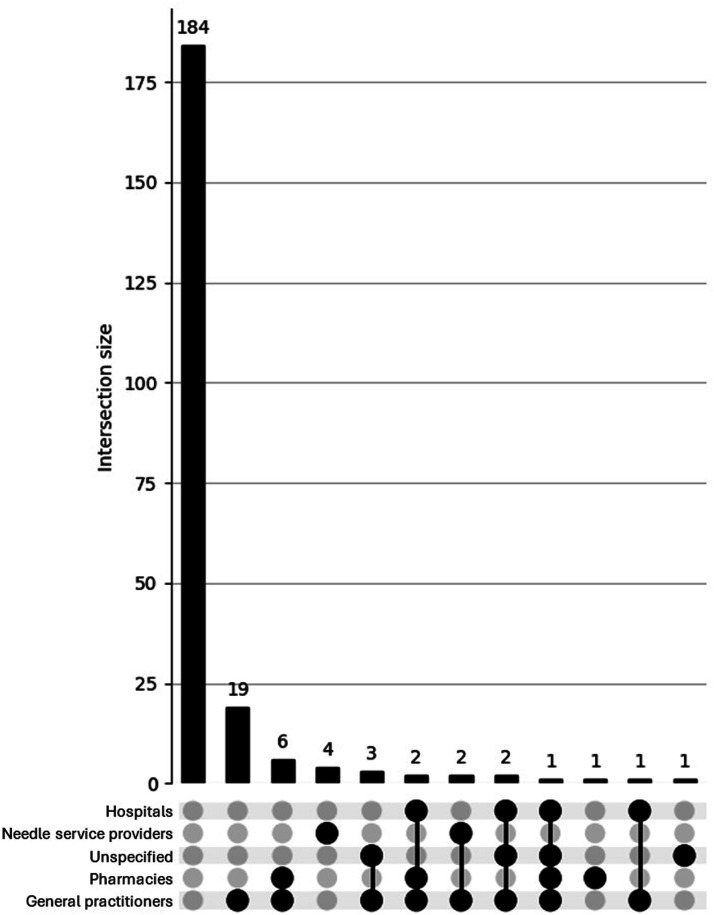
UpSet plot depicting access difficulties participants (n = 226) aged *≥*40.

The UpSet plot for the<40 group (see Figure 5) reveals a broader and more complex pattern of access difficulties. Although most participants reported no issues, a substantial proportion endorsed overlapping difficulties across services such as general practitioners, hospitals, pharmacies, and needle providers. Several multi-category intersections involved more than 10 participants, indicating a diverse set of challenges in accessing health care while using AAS. In contrast, the UpSet plot for the *≥*40 group (see Figure 6) shows a more limited distribution of difficulties. Most older participants reported either no issues or a single isolated difficulty, with only minimal multi-service overlap observed. This suggests that access barriers among older men who use AAS are more likely to be specific and compartmentalised, rather than part of a broader pattern of systemic difficulty. Overall, the findings highlight a greater complexity of health care access issues among younger men who use AAS.

### Inferential Statistics: Group Comparisons

#### Psychosocial Concerns

Chi-square analyses indicated that the <40 group was significantly more likely than the *≥*40 group to report anger/aggression (*χ*^*2*^ [1] = 4.74, *p* = .029, *ϕ* = .064), depression/low mood (*χ*^*2*^ [1] = 4.07, *p* = .044, *ϕ* = .060), rapid mood fluctuations (*χ*^*2*^ [1] = 5.08, *p* = .024, *ϕ* = .067), restlessness/irritability (*χ*^*2*^ [1] = 6.52, *p* = .011, *ϕ* = .075), and loss of interest in other things (*χ*^*2*^ [1] = 5.83, *p* = .016, *ϕ* = .071). All effect sizes were small in magnitude. No significant group differences emerged for irrational excitability, quality of relationships, or other psychosocial concerns (all *p*s > .057; *ϕ*s < .06). These results suggest a broader but modestly elevated psychosocial burden among younger participants.

#### Physical Concerns

Chi-square analyses indicated that the <40 group was significantly more likely than the *≥*40 group to report hair loss (*χ*^*2*^ [1] = 8.79, *p* = .003, *ϕ* = .088), decreased fertility (*χ*^*2*^ [1] = 31.13, *p* < .001, *ϕ* = .165), and skin conditions (e.g. acne; *χ*^*2*^ [1] = 6.40, *p* = .011, *ϕ* = .075). These effect sizes ranged from small to moderate in magnitude. No significant differences were observed for other physical concerns, including decreased sexual function, hair gain, negative impact on sexual organs, gynaecomastia, changes in breast size, negative impact on the heart or liver, and other concerns (all *p*s > .050). These findings suggest that specific physical side effects may be more prevalent among younger men who use AAS, highlighting key areas for age-targeted harm reduction.

#### Access Difficulties

Chi-square analyses indicated that the <40 group was significantly more likely than the*≥*40 group to report difficulties accessing hospitals (*χ*^*2*^ [1] = 5.64, *p* = .018, *ϕ* = .07) and pharmacies when required (*χ*^*2*^ [1] = 4.03, *p* = .045, *ϕ* = .06). Both effect sizes were small. Specifically, 6.8% of the <40 group reported difficulty accessing hospitals compared to 2.7% of the *≥*40 group; 8.4% of the <40 group reported difficulty accessing pharmacies compared to 4.4% of the *≥*40 group. No significant group differences were observed for difficulties accessing needle service providers, general practitioners, or other services (all *p*s > .089). These results suggest that younger men who use AAS may face more difficulty accessing certain health care services, though the differences are modest.

## Discussion

The present study aimed to discern differences, between younger (<40) and older (≥40) men who use AAS, in self-reported co-occurrences of physical concerns, psychosocial concerns, and access to health services. The findings revealed a clear divergence in the risk profiles between these 2 groups. Firstly, with regards to psychosocial concerns, younger men were significantly more likely to report a range of adverse emotional and behavioural outcomes, including anger/aggression, depression, rapid mood fluctuation, restlessness, and loss of interest in other activities. While effect sizes were small, the consistency across multiple dimensions suggests a meaningful psychosocial burden among younger men who use AAS. This is reinforced by the data visualisation, which showed denser, more complex patterns of symptom co-occurrence in the younger cohort, with numerous intersecting concerns. In contrast, older men displayed a flatter psychosocial distribution, with most individuals reporting no concerns or only isolated symptoms. Secondly, with regards to physical concerns, younger men who use AAS, again, reported significantly higher rates of specific issues, including hair loss, decreased fertility, and skin conditions. Data visualisation further underscored this difference, whereby younger participants showed a broader range of overlapping concerns, with multiple high frequency intersections suggesting a more heavily clustered physical side-effect profile. Older men who use AAS, by comparison, exhibited simpler profiles with fewer overlapping concerns.

This divergence between AAS use age-groups may be partly explained by differential access to regulated compound sourcing and clinical guidance.^10,11^ As described earlier, younger men who use AAS often rely on informal peer advice, variable-quality compounds, and unstructured usage strategies, and these conditions that may elevate both physical and psychosocial risk.^11,27,28^ Meanwhile, older men who use AAS, particularly those entering via therapeutic routes, may benefit from consistent dosing, medically supervised use, and greater health literacy.^12,14,32^ Together, these patterns suggest that younger men who use AAS are operating under conditions of both higher exposure and lower support. For instance, the denser psychosocial and physical overlap observed in younger men who use AAS likely reflects a broader risk profile shaped by differing motivations, limited medical oversight, and more informal access pathways.^7,8,11^ Compared to older men who use AAS, whose use is more often embedded in clinical settings, younger men who use AAS appear more exposed to harm due to peer-led practices, inconsistent compound quality, and a lack of structured guidance.^27,28^ Where older men who use AAS may be incrementally adopting enhancement motives traditionally associated with younger men, the reverse does not appear to be true in terms of harm mitigation. For instance, there is no evidence that younger men who use AAS are gaining the structural protections historically afforded to older – medically supervised – men who use AAS.^9,54^ This imbalance positions younger men who use AAS as a particularly high-risk group, not simply due to biological vulnerability, but due to the ecosystem of unsupervised and unregulated practices in which they operate.^11,28,54^ These findings provide a novel contribution to this rapidly emerging literature corpus, by quantitatively mapping how harm profiles differ by age, offering a sharper lens for understanding risk pathways within an increasingly heterogeneous AAS-using population.

The data also demonstrated age-related differences in access to health care services. While over half of all participants reported at least 1 barrier to timely or appropriate access, younger men who use AAS were significantly more likely to report problems accessing care through pharmacies and hospitals. Data visualisations supported this disparity, revealing a broader and more complex pattern of service-related barriers among younger men who use AAS, often involving multiple access points. In contrast, access difficulties among older participants tended to be more isolated and less severe. Again, these findings reflect the structural consequences of use for all people who use AAS.^30^ However, particularly for younger men, whose enhancement goals often lack medical legitimacy in the eyes of health care providers, may be met with greater resistance or stigma when attempting to engage with formal systems.^29-31^ Conversely, older men who use AAS may be more likely to receive prescriptions or assistance from clinicians, particularly when presenting with age-related testosterone decline.^12,14,32^ This divergence not only reinforces the exclusionary dynamics outlined earlier, but highlights access itself as a critical harm determinant, shaping where, how, and whether individuals receive appropriate care, safe compounds, or credible harm reduction advice.

The findings of this study have important implications for harm reduction and health service design targeting men who use AAS. Most notably, they highlight the need for differentiated, age-responsive strategies. Current models of AAS-related harm reduction may inadequately address the specific needs of younger men, who are more likely to operate outside clinical frameworks and encounter structural barriers to care. Interventions must, therefore, extend beyond generalised messaging and consider the informal, peer-driven contexts in which younger men often initiate and maintain AAS use. This includes improving the reach and credibility of harm reduction messaging within online and peer spaces,^55-57^ as well as addressing the stigma that deters help-seeking. Further compounding these issues is the ‘othering’ that occurs within AAS-using communities, between people who use AAS and other communities of people who use drugs. This means that information communicated in places where all substance use may be present (e.g. needle exchanges, pharmacies) may not be optimal when considering aspects of cultural safety and relevance. For instance, any information related to AAS disseminated at these sites (e.g. needle exchanges) may not receive any buy-in and may be contributing to the conflation of access issues seen among our international sample. Therefore, further research and consideration is warranted regarding the way local contexts and cultural safety interact with age-specific factors related to discussions and resources aimed to reduce AAS-related harms.

In contrast, older men who use AAS may benefit from resources that bridge therapeutic and enhancement discourses, ensuring continued harm minimisation even within clinically facilitated use.^58^ However, older men who use AAS are also more likely to trust their health professionals and, taken together with their lack of self-reported harms as seen in this study, make them optimal candidates for healthy lifestyle interventions which bring together androgens, exercise, and dietary elements. Therefore, health services should also be equipped to distinguish between age-related entry pathways, offering tailored support that acknowledges varying motivations, use patterns, and structural vulnerabilities. These insights reinforce the value of an intersectional, life-course approach to AAS policy and practice.

### Limitations

The present study offers several methodological strengths. The use of GDS2024 data enabled access to a large international convenience sample (N = 1146) of men who reported AAS consumption within the past 12 months, a task that is difficult to achieve through traditional probability sampling.^46^ The online, anonymous, and unincentivised nature of the survey is also well-suited to collecting sensitive self-report data, particularly in relation to illicit or stigmatised health behaviours.^59^ Moreover, recent work supports the psychometric robustness of self-reported substance use data, particularly when collected under conditions like those employed by the GDS2024.^60^ Finally, this study introduced a novel visualisation approach using UpSet plots to clarify the complexity and co-occurrence of harm profiles within and across age groups, offering a unique methodological contribution to the field.

Despite these strengths, several limitations should be acknowledged. First, the cross-sectional design precludes causal inference, as the temporal ordering between AAS consumption patterns and reported harms cannot be established. Second, while the sample was relatively large, it was recruited via convenience sampling and may not fully represent the broader AAS population. For example, the sample skewed heavily toward White participants (78.1%) and individuals with post-secondary education (57.2%), which may limit generalisability to more diverse or underrepresented groups. Third, all data were self-reported, which may introduce recall bias or social desirability effects, although the anonymity of the survey likely reduced these concerns. Fourth, concerns were assessed using dichotomous yes/no items rather than graded response scales. This approach reflects established practice across prior GDS waves^46^ and GDS-based AAS/IPED analyses,^49,50^ where binary indicators maximise respondent comprehension across languages, minimise survey burden, and reliably capture the presence vs absence of harms in large community samples. While this enhances clarity for participants at the point of data collection, it unavoidably limits the level of detail available for statistical analysis. Fifth, the survey did not capture participants’ sources of information about AAS use, which could have offered valuable context for interpreting age-related differences. Sixth, health care access varies substantially across national systems (e.g. universal coverage vs private insurance models), which may have influenced participants’ reported experiences of access barriers. Because the sample was international and convenience-based, we were not able to isolate or control for these contextual differences. Seventh, legal frameworks surrounding AAS use differ markedly across countries, from prohibition to medical regulation (see Piatkowski et al, 2024 [57]). These differences likely influenced participants’ access pathways and willingness to seek information or health care, which we could not isolate in the present study.

### Conclusions

This study contributes new evidence to the literature on AAS-related harm by delineating how psychosocial concerns, physical concerns, and access difficulties vary across younger and older men. Findings indicate that younger men who use AAS face a denser burden of co-occurring harms, likely shaped by non-medical motivations, reduced clinical oversight, and more informal access pathways. In contrast, older men who use AAS tended to report fewer and less complex harms, potentially reflecting safer compound sourcing, and greater integration with health care systems. These findings highlight the need for contextually age-appropriate harm reduction strategies that recognise the diverse health service needs, and structural vulnerabilities of younger and older men who use AAS.

## Consent to Participate

This study involved secondary analysis of fully anonymised data.

## Consent for Publication

No identifying participant information is included in this article.

## Data Availability

The data that support the findings of this study are not publicly available due to privacy and governance restrictions but may be available upon reasonable request.*

## References

[1] Horwitz H Andersen J Dalhoff K . Health consequences of androgenic anabolic steroid use. J Intern Med. 2019;285(3):333-340. 30460728 10.1111/joim.12850

[2] Grant B Hyams E Davies R Minhas S Jayasena CN . Androgen abuse: risks and adverse effects in men. Ann N Y Acad Sci. 2024;1538(1):56-70. 39041466 10.1111/nyas.15187

[3] Sagoe D Molde H Andreassen CS Torsheim T Pallesen S . The global epidemiology of anabolic-androgenic steroid use: a meta-analysis and meta-regression analysis. Ann Epidemiol. 2014;24(5):383-398. 24582699 10.1016/j.annepidem.2014.01.009

[4] Piatkowski T Whiteside B Robertson J Henning A Lau EH Dunn M . What is the prevalence of Anabolic‐Androgenic steroid use among women? A systematic review. Addiction. 2024;119(12):2088-2100. 39134450 10.1111/add.16643

[5] Bhasin S Brito JP Cunningham GR , et al. Testosterone therapy in men with hypogonadism: an endocrine society clinical practice guideline. J Clin Endocrinol Metab. 2018;103(5):1715-1744. 29562364 10.1210/jc.2018-00229

[6] Woerdeman J de Ronde W . Therapeutic effects of anabolic androgenic steroids on chronic diseases associated with muscle wasting. Expet Opin Invest Drugs. 2011;20(1):87-97. 10.1517/13543784.2011.544651 21158691

[7] Kanayama G Hudson JI Pope JHG . Anabolic-androgenic steroid use and body image in men: a growing concern for Clinicians. Psychother Psychosom. 2020;89(2):65-73. 32066136 10.1159/000505978

[8] Santos GH Coomber R . The risk environment of anabolic–androgenic steroid users in the UK: examining motivations, practices and accounts of use. Int J Drug Pol. 2017;40:35-43. 10.1016/j.drugpo.2016.11.005 27955960

[9] Gestsdottir S Kristjansdottir H Sigurdsson H Sigfusdottir ID . Prevalence, mental health and substance use of anabolic steroid users: a population-based study on young individuals. Scand J Publ Health. 2021;49(5):555-562. 10.1177/1403494820973096 33280527

[10] Dunn M Piatkowski T . Investigating the impact of COVID-19 on performance and image enhancing drug use. Harm Reduct J. 2021;18(1):1-8. 34863199 10.1186/s12954-021-00571-8 PMC8642842

[11] Turnock L Gibbs N Cox L Piatkowski T . Big business: the private sector market for image and performance enhancing drug harm reduction in the UK. Int J Drug Pol. 2023;122:104254. 10.1016/j.drugpo.2023.104254 37950942

[12] Dunn M Mulrooney KJ Forlini C van de Ven K Underwood M . The pharmaceuticalisation of ‘healthy’ageing: testosterone enhancement for longevity. Int J Drug Pol. 2021;95:103159. 10.1016/j.drugpo.2021.103159 33583680

[13] Hoberman JM Yesalis CE . The history of synthetic testosterone. Sci Am. 1995;272(2):76-81. 10.1038/scientificamerican0295-76 7817189

[14] Yeap BB Tran C Douglass CM McNeil JJ . Testosterone therapy in older men: present and future considerations. Drugs Aging. 2025;42:1-12. 40287898 10.1007/s40266-025-01209-1 PMC12148964

[15] Underwood M van de Ven K Dunn M . Testing the boundaries: self-Medicated testosterone replacement and why it is practised. Int J Drug Pol. 2021;95:103087. 10.1016/j.drugpo.2020.103087 33342615

[16] Handelsman DJ . Global trends in testosterone prescribing, 2000–2011: expanding the spectrum of prescription drug misuse. Med J Aust. 2013;199(8):548-551. 24138381 10.5694/mja13.10111

[17] Hearne E Atkinson A Boardley I McVeigh J Van Hout MC . Sustaining masculinity’: a scoping review of anabolic androgenic steroid use by older males. Drugs Educ Prev Pol. 2024;31(1):27-53.

[18] Leone JE Fetro JV . Perceptions and attitudes toward androgenic-anabolic steroid use among two age categories: aqualitative inquiry. J Strength Condit Res. 2007;21(2):532-537. 10.1519/R-18665.1 17530945

[19] Albano GD Amico F Cocimano G , et al., eds. Adverse Effects of anabolic-androgenic Steroids: A Literature Review. Healthcare. MDPI; 2021. 10.3390/healthcare9010097 PMC7832337 33477800

[20] Abdullah R Bjørnebekk A Hauger LE , et al. Severe biventricular cardiomyopathy in both current and former long-term users of anabolic–androgenic steroids. Eur J Prev Cardiol. 2024;31(5):599-608. 37992194 10.1093/eurjpc/zwad362

[21] Armstrong JM Avant RA Charchenko CM , et al. Impact of anabolic androgenic steroids on sexual function. Transl Androl Urol. 2018;7(3):483-489. 30050806 10.21037/tau.2018.04.23 PMC6043738

[22] Neupane S Kalra F . Association of anabolic steroid use with hypertension and cardiomyopathy: a case study. Cureus. 2024;16(10):e71775. 39559620 10.7759/cureus.71775 PMC11570439

[23] Karagun B Altug S . Anabolic-androgenic steroids are linked to depression and anxiety in Male bodybuilders: the hidden psychogenic side of anabolic androgenic steroids. Ann Med. 2024;56(1):2337717. 38590148 10.1080/07853890.2024.2337717 PMC11005876

[24] Piatkowski T De Andrade D Neumann D Tisdale C Dunn M . Examining the association between trenbolone, psychological distress, and aggression among males who use anabolic-androgenic steroids. Int J Drug Pol. 2024;134:104636. 10.1016/j.drugpo.2024.104636 39486244

[25] Scarth M Havnes IA Jørstad ML Bjørnebekk A . Psychological traits associated with anabolic androgenic steroid use and dependence among female athletes. BMC Womens Health. 2023;25(1):214. 10.1186/s12905-025-03711-5 PMC12051273 40325431

[26] Underwood M . From ‘bro, do you even lift?’to ‘bro, do you Even science?’: how the relationship between science and broscience can inform the development of allied image and performance enhancing drug harm reduction. Performance Enhancement & Health. 2025;13(1):100291.

[27] Henning A Andreasson J . Preventing, producing, or reducing harm? Fitness doping risk and enabling environments. Drugs Educ Prev Pol. 2022;29(1):95-104.

[28] Piatkowski T Gibbs N Neumann D Dunn M . Consuming ‘God Juice’: using ‘ethnopharmacological‐connoisseurship’to situate trenbolone use and knowledge among image and performance enhancing drug communities. Drug Alcohol Rev. 2025;44(1):298-309. 39433468 10.1111/dar.13965

[29] Piatkowski T Ayurzana L King M Hattingh L McMillan S . Community pharmacy’s role in dispensing androgens and supporting harm reduction amid current policy dilemmas. Subst Abuse Treat Prev Pol. 2025;20(1):1-12. 10.1186/s13011-025-00636-y PMC11748596 39827172

[30] Cox L Piatkowski T McVeigh J . “I would never go to the doctor and speak about steroids”: anabolic androgenic steroids, stigma and harm. Drugs Educ Prev Pol. 2024;32:1-13.

[31] Piatkowski T Benn S King M McMillan S Hattingh L . Pharmacies are less confronting than a medical practitioner. In: A Qualitative Exploration of Community Pharmacy as an Environment for Reducing Harms Related to anabolic-androgenic Steroid Use; 2023.

[32] Yabluchanskiy A Tsitouras PD . Is testosterone replacement therapy in older men effective and safe? Drugs Aging. 2019;36(11):981-989. 31595418 10.1007/s40266-019-00716-2 PMC8596965

[33] Tenover JL . Testosterone replacement therapy in older adult men. Int J Androl. 1999;22(5):300-306. 10509230 10.1046/j.1365-2605.1999.00184.x

[34] de Zeeuw TI Brunt TM van Amsterdam J van de Ven K van den Brink W . Anabolic androgenic steroid use patterns and steroid use disorders in a sample of Male gym visitors. Eur Addctn Res. 2023;29(2):99-108. 10.1159/000528256 PMC10273855 36731448

[35] Piatkowski T Coomber R Francis C , et al. The world's first anabolic‐androgenic steroid testing trial: a two‐phase pilot combining chemical analysis, results dissemination and community feedback. Addiction. 2025;120(7):1366-1377. 39911049 10.1111/add.70009 PMC12128565

[36] Rowe R Berger I Yaseen B Copeland J . Risk and blood‐borne virus testing among men who inject image and performance enhancing drugs, Sydney, Australia. Drug Alcohol Rev. 2017;36(5):658-666. 28244160 10.1111/dar.12467

[37] Rich J Dickinson B Feller A Pugatch D Mylonakis E . The infectious complications of anabolic-androgenic steroid injection. Int J Sports Med. 1999;20(08):563-566. 10606223 10.1055/s-1999-8841

[38] Havnes IA Jørstad ML Wisløff C . Anabolic-androgenic steroid users receiving health-related information; health problems, motivations to quit and treatment desires. Subst Abuse Treat Prev Pol. 2019;14:1-12. 10.1186/s13011-019-0206-5 PMC6524231 31096999

[39] Bjørnebekk A Westlye LT Walhovd KB Jørstad ML Sundseth ØØ Fjell AM . Cognitive performance and structural brain correlates in long-term anabolic-androgenic steroid exposed and nonexposed weightlifters. Neuropsychology. 2019;33(4):547-559. 31033318 10.1037/neu0000537

[40] Kim M Suh D Barone JA Jung S-Y Wu W Suh D-C . Health literacy level and comprehension of prescription and nonprescription drug information. Int J Environ Res Publ Health. 2022;19(11):6665. 10.3390/ijerph19116665 PMC9180079 35682249

[41] Andreasson J Johansson T . Online doping. The new self-help culture of ethnopharmacology. Sport Soc. 2016;19(7):957-972.

[42] Kelsey TW Li LQ Mitchell RT Whelan A Anderson RA Wallace WHB . A validated age-related normative model for Male total testosterone shows increasing variance but no decline after age 40 years. PLoS One. 2014;9(10):e109346. 25295520 10.1371/journal.pone.0109346 PMC4190174

[43] Piatkowski T Havnes IA Kill E van de Ven K . Could Testosterone Be the New Methadone? New Ways for Approaching anabolic-androgenic Steroid Dependence. Elsevier; 2024:100275.

[44] Yeap BB Grossmann M McLachlan RI , et al. Endocrine society of Australia position statement on Male hypogonadism (part 1): assessment and indications for testosterone therapy. Med J Aust. 2016;205(4):173-178. 27510348 10.5694/mja16.00393

[45] Stanworth RD Jones TH . Testosterone for the aging Male; current evidence and recommended practice. Clin Interv Aging. 2008;3(1):25-44. 18488876 10.2147/cia.s190 PMC2544367

[46] Barratt MJ Ferris JA Zahnow R Palamar JJ Maier LJ Winstock AR . Moving on from representativeness: testing the utility of the global drug survey. Subst Abuse. 2017;11:1178221817716391. 28924351 10.1177/1178221817716391 PMC5595253

[47] Winstock AR Davies EL Ferris JA Maier LJ Barratt MJ . Using the global drug survey for harm reduction. Monitoring drug use in the digital age: Studies in web surveys. 2022;26.

[48] Winstock AR Barratt MJ Maier LJ , et al. Global drug survey 2019: key findings report. 2020.

[49] Bonenti B Puljević C Steve V , et al. Clenbuterol and the cost of cutting: a brief report comparing self-reported side effects of clenbuterol consumption to anabolic-androgenic steroid compounds. Performance Enhancement & Health. 2025;13(4):100356.

[50] Grobler E Puljević C Bonenti B , et al. Associations between anabolic-androgenic steroid testing, healthcare access and undesirable effects among international consumers. Performance Enhancement & Health. 2026;14(1):100390.

[51] IBM Corp. IBM SPSS Statistics for Windows, Version 30.0. Armonk (NY): IBM Corp; 2024.

[52] Python Software Foundation. Python: Version 3.12.0. Python Software Foundation; 2024.

[53] Cohen J . The effect size. In: Statistical Power Analysis for the Behavioral Sciences. Abingdon: Routledge; 1988:77-83.

[54] Underwood M . Exploring the social lives of image and performance enhancing drugs: an online ethnography of the zyzz fandom of recreational bodybuilders. Int J Drug Pol. 2017;39:78-85. 10.1016/j.drugpo.2016.08.012 27768997

[55] Piatkowski T Akrigg K Cox L Bradshaw A Vigorous S . Anything but androgens: how image and performance enhancing drug consumers manage body composition and health through off-label use of medicines. Performance Enhancement & Health. 2025;13:100329.

[56] Turnock LA Townshend HD . How digital fitness forums shape IPED access, use, and community harm reduction behaviours. In: Doping in Sport and Fitness. Emerald Publishing Limited; 2022:155-179.

[57] Turnock L . Outlining a typology of steroid suppliers located on a popular international fitness and bodybuilding forum. Kriminol J. 2021;53(3).

[58] Magnolini R Kossinna K Bjasch D Kruijver M Bruggmann P Senn O . Feasibility of implementing current best clinical practice for people who are using anabolic androgenic steroids within a Swiss primary care practice: a quality assurance study. Swiss Med Wkly. 2025;155(2):4225. 39977451 10.57187/s.4225

[59] Kays KM Keith TL Broughal MT . Best practice in online survey research with sensitive topics. Advancing research methods with new technologies: IGI Global. 2013;157-168.

[60] Bharat C Degenhardt L Pearson SA , et al. A data‐informed approach using individualised dispensing patterns to estimate medicine exposure periods and dose from pharmaceutical claims data. Pharmacoepidemiol Drug Saf. 2023;32(3):352-365. 36345837 10.1002/pds.5567 PMC10947320

